# Dementia and all‐cause mortality in older adults: Findings from the ELSI‐Brazil study

**DOI:** 10.1002/alz.71400

**Published:** 2026-05-06

**Authors:** Matheus Ghossain Barbosa, Lucas Martins Teixeira, Juliana Vaz de Melo Mambrini, Wendell Lima Rabelo, Andrew Christopher Claro Miguel, Maria Fernanda Lima‐Costa, Cleusa Pinheiro Ferri

**Affiliations:** ^1^ Department of Psychiatry, Psychogeriatric Unit, Escola Paulista de Medicina Universidade Federal de São Paulo São Paulo Brazil; ^2^ Responsabilidade Social, Hospital Alemão Oswaldo Cruz São Paulo Brazil; ^3^ René Rachou Research Institute Fundação Oswaldo Cruz Belo Horizonte Brazil; ^4^ Medical School Universidade Federal de Minas Gerais, Post‐Graduation in Public Health Belo Horizonte Brazil

**Keywords:** Brazil, cognition, dementia, functionality, LMICs, mortality

## Abstract

**INTRODUCTION:**

Although nearly two‐thirds of all dementia cases occur in low‐ and middle‐income countries, evidence on dementia‐related mortality in these settings remains scarce.

**METHODS:**

We analyzed 5249 participants aged ≥60 years from the Brazilian Longitudinal Study of Aging (ELSI‐Brazil). Dementia was defined using an algorithm or self‐reported diagnosis. Mortality rates were estimated by dementia status, sex, and age. Among individuals with dementia (*n* = 392), multivariate Cox models assessed predictors of all‐cause mortality.

**RESULTS:**

Mortality was higher in individuals with dementia even after adjusting for age and sex (hazard ratio: 2.65; 95% confidence interval: 2.08 to 3.39). Among people with dementia, male sex, higher education, poorer functional status, and lower cognitive performance increased mortality risk, while having a partner was protective.

**DISCUSSION:**

Dementia markedly increased mortality risk. Functional impairment, cognitive decline, social support, and schooling were key determinants of survival, emphasizing the importance of targeted, context‐specific strategies to improve outcomes for people living with dementia.

## Highlights

- Data on dementia‐related mortality are scarce in LMICs.
- This study presents the first national representative dementia‐related mortality estimates for Brazil.
- Age and sex adjusted mortality risk was 2.6 times higher among people with dementia.
- Male sex, higher education, poor function, and worse cognition predicted mortality.
- Having a partner was protective against mortality.

## 1 BACKGROUND

Dementia is a major cause of disability and dependence among older adults and represents a significant global public health challenge. In 2023, it was the sixth leading cause of death worldwide and the third in Brazil.^1^ According to the Global Burden of Disease (GBD) Study, 57.4 million people were living with the condition in 2019, a number projected to reach 152.8 million by 2050 due to rapid population aging.^2^ It is characterized by progressive cognitive decline, behavioral disturbances, and loss of functional independence, ultimately increasing mortality risk, often through respiratory complications such as bronchopneumonia, commonly precipitated by feeding difficulties.^3^

More than 60% of people living with dementia reside in low‐ and middle‐income countries (LMICs), a proportion projected to rise to 71% by 2050.^4^ In Brazil, an estimated 2.5 million individuals had dementia in 2019, with projections reaching 5.4 million by 2040.^5^ The economic burden in the country is substantial: Total costs were estimated at $35.3 billion in 2019, 73% of which were indirect costs related to unpaid caregiving and productivity losses.^6^ These challenges unfold within a health system that already faces significant constraints in providing adequate care for older adults, and the burden is expected to grow as life expectancy rises.^2^

Several studies have examined dementia‐related mortality globally. Mortality risk in individuals with dementia is consistently higher than in the general population, with median survival ranging from 3 to 12 years after symptom onset and 3 to 7 years after diagnosis.^7^ Factors associated with increased mortality risk include older age, poorer cognition, male sex, diabetes, stroke, cardiovascular disease, smoking, multimorbidity, and polypharmacy,^8^, ^9^, ^10^ and survival estimates vary widely across populations.^11^, ^12^

In LMICs, mortality hazard ratios (HRs) for dementia range from 1.6 to 5.7 when compared with cognitively healthy older adults,^12^ and substantial heterogeneity in risk factor assessment has been reported.^7^ The 10/66 Dementia Research Group, which investigates dementia in LMIC contexts, has identified several factors associated with mortality, including older age, male sex, poorer cognition, reduced functional capacity, and undernutrition.^13^

Particularly in Brazil, however, evidence on dementia‐related mortality remains scarce. Although three population‐based studies have investigated risk factors for dementia incidence,^14^, ^15^, ^16^ only one of them – conducted two decades ago in a single municipality – has examined mortality determinants, reporting a mortality risk ratio 3.9 times higher among individuals with dementia compared with others in the same age group.^17^ Given the country's demographic, socioeconomic, and regional diversity, nationally representative data are essential to better understand mortality patterns among older adults with dementia.

Factors such as reduced access to healthcare, lower educational attainment, and socioeconomic inequalities may influence dementia outcomes in LMIC contexts. These elements, combined with regional disparities, highlight the need for locally relevant evidence to inform policies and improve care strategies. This study aims to address this gap by estimating mortality rates and identifying factors associated with increased mortality among older Brazilians living with dementia, using nationally representative data from the Brazilian Longitudinal Study of Aging (ELSI‐Brazil).

## 2 METHODS

### 2.1 Study design and population

This study is a population‐based cohort analysis using data from ELSI‐Brazil, a nationally representative survey of community‐dwelling adults aged ≥50 years and an international partner study of the Health and Retirement Study (HRS).^18^ All residents aged ≥50 years within sampled households were eligible for individual interviews and physical assessments. In households with more than one eligible adult, residents selected one respondent for the household questionnaire. Baseline data were collected in 2015 to 2016 across 70 municipalities in the five Brazilian geopolitical regions using standardized, home‐based assessments conducted by trained interviewers. These assessments included sociodemographic characteristics, lifestyle factors, mental and physical health measures, healthcare utilization, and physical performance tests. Of the 10,000 planned interviews, 9412 were completed.

Detailed descriptions of sampling, weighting procedures, and operational methods have been published elsewhere.^19^, ^20^

**RESEARCH IN CONTEXT**

1. **Systematic review**: The authors reviewed the literature related to dementia, mortality, and LMICs. Evidence on dementia‐related mortality in LMICs is limited, with Brazil having only one previous population‐based study conducted over two decades ago. No prior study provided nationally representative mortality estimates in Brazil.
2. **Interpretation**: This study shows that all‐cause mortality is substantially increased among participants with dementia in a large, nationally representative Brazilian cohort. Functional impairment, lower cognitive performance, male sex, and higher educational attainment were independent predictors of increased mortality, while having a partner was protective.
3. **Future directions**: Future studies should investigate cause‐specific mortality in dementia, explore regional and socioeconomic disparities in survival across Brazil, and evaluate whether interventions aimed at maintaining functional capacity, improving physical activity levels, and enhancing early detection of cognitive decline can reduce mortality among older adults with dementia.

**FIGURE 1:**
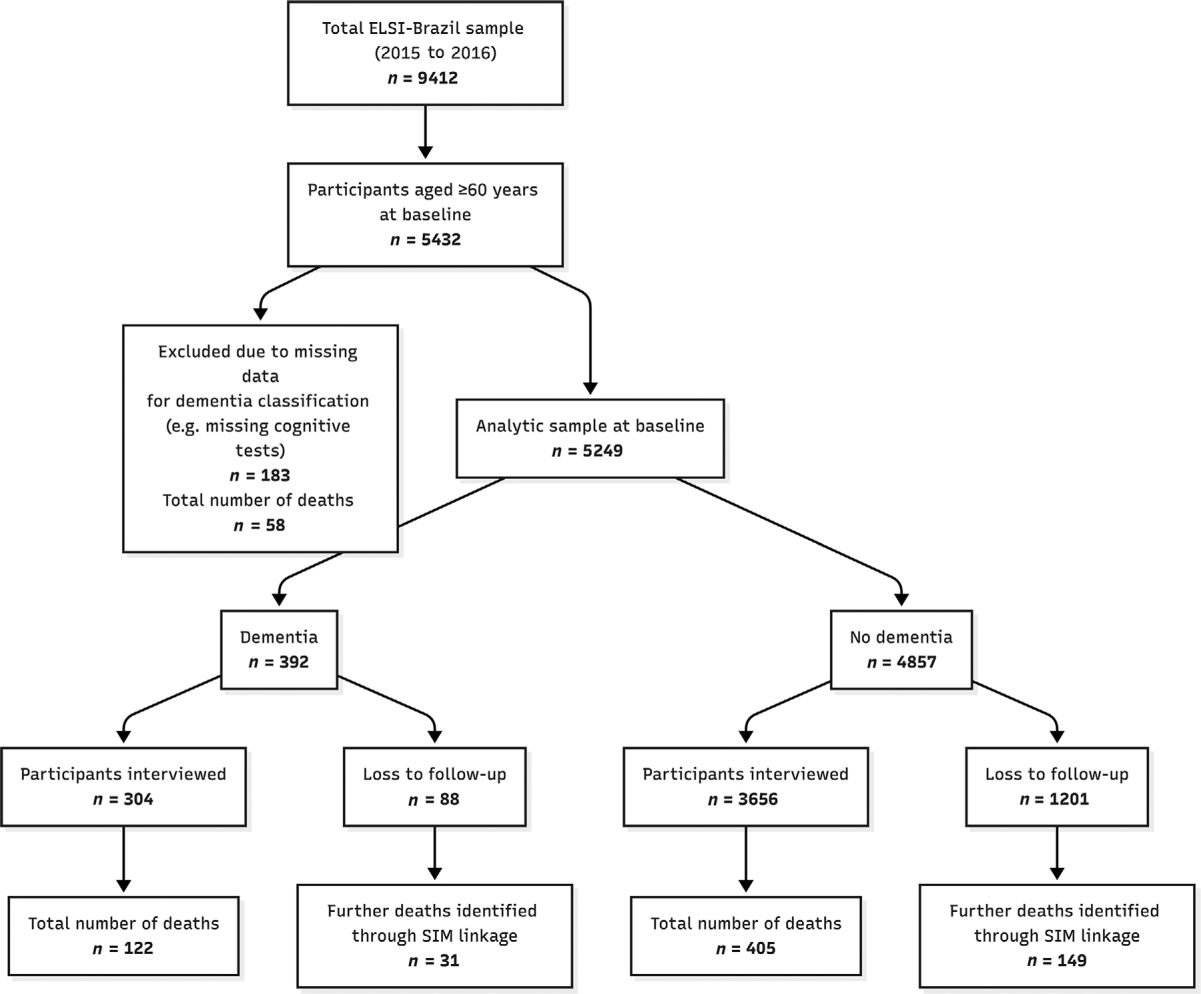
Participant flow diagram. Selection of analytic sample from Brazil Longitudinal Study of Aging baseline cohort (2015 to 2016) and follow‐up for mortality outcome (2019 to 2021). IADL: Instrumental Activities of Daily Living, IQCODE: Informant Questionnaire on Cognitive Decline in the Elderly, SIM: Sistema de Informações sobre Mortalidade (Mortality Information System).

The final sample was restricted to 5249 people aged 60 and above for whom a dementia ascertainment was possible at baseline. Of the 5432 participants aged 60 years and older at baseline, 183 (3.4%) had missing data on key variables required for dementia ascertainment (including cognitive assessment, Instrumental Activities of Daily Living [IADL] items and the Informant Questionnaire on Cognitive Decline in the Elderly [IQCODE]) and were therefore not included in the final sample. Vital status was ascertained during the second wave (2019 to 2021). A flowchart detailing participant selection, exclusions, and follow‐up is presented in Figure 1.

### 2.2 Measures

#### 2.2.1 Dementia classification

Dementia status was determined using a previously published algorithm,^21^ combined with self‐reported diagnosis of Alzheimer's disease. The algorithm was based on the cognitive assessments conducted as part of ELSI‐Brazil, which included temporal orientation (day, month, year, and weekday), semantic verbal fluency (1‐min animal naming), a 10‐word list learning test (immediate and delayed recall), prospective memory (delayed instruction recall), and four semantic memory items (two related to everyday knowledge and two related to political knowledge).

Five cognitive domain scores (orientation, verbal fluency, episodic memory, semantic memory, and prospective memory) were standardized into *z*‐scores; their mean constituted the global cognitive score.

A normative subsample was derived by excluding participants with: (1) visual or hearing impairments affecting test performance; (2) previous depression diagnosis or depressive symptoms (CES‐D ≥ 4)^22^; (3) history of Alzheimer's disease, Parkinson's disease, or stroke (self‐report or informant report); (4) heavy drinking, per National Institute on Alcohol Abuse and Alcoholism criteria^23^; (5) memory complaints (self or informant report); (6) impairment in at least one of four gender‐independent IADLs (managing money, using transportation, using the telephone, and taking medications); (7) missing cognitive or exclusion‐criteria data. After excluding individuals who did not meet these criteria, of 5432 participants aged 60 years or older in the initial sample, the normative subsample comprised 908 individuals.

Regression‐based norms were generated to predict global cognitive scores based on age, education, and sex. Residual scores (observed−predicted, standardized by the normative residual standard deviation [SD]) were then produced. A residual global cognitive *z*‐score ≤ −1.5 defined cognitive impairment. Participants meeting this threshold who also had functional impairment in at least one of the four IADLs or an IQCODE score ≥ 3.4 were classified as having dementia. Full procedures are described elsewhere.^21^

Participants who self‐reported a previous diagnosis of Alzheimer's disease were also classified as having dementia, regardless of the algorithm results.

#### 2.2.2 Mortality data

Mortality information was obtained during the second wave (2019 to 2021) using proxy respondent reports and linkage with Brazil's national Mortality Information System (Sistema de Informações sobre Mortalidade [SIM]). Participants who were not confirmed as deceased were considered alive, and a census was conducted on March 21, 2021. During the follow‐up period (2015/2016 to 2019/2021) with a total of 26,042.8 person‐years, 707 deaths occurred among the 5249 included participants (Figure 1), with 527 being identified during interviews (122 among people with dementia and 405 among people without dementia). Cross checking with SIM identified an additional 180 additional deaths (31 among people with dementia and 149 among people without dementia) among the 1109 participants lost to follow‐up.

#### 2.2.3 Other measures

Sociodemographic and health‐related variables were included as potential predictors of mortality. These measures comprised age, sex, marital status (having or not having a partner), skin color, place of residence (urban vs rural), years of education, number of chronic conditions, recent use of health services, current smoking, current alcohol consumption, insufficient physical activity, cognitive performance, and functional status.

The total number of chronic conditions was derived from self‐reported medical diagnoses of 14 conditions: hypertension, diabetes, high cholesterol, myocardial infarction, angina, heart failure, stroke, asthma, chronic obstructive pulmonary disease, arthritis, osteoporosis, chronic back pain, cancer, and chronic kidney disease. Access to health services was assessed through self‐reported use of any healthcare service (including family doctor or specialist consultations, emergency department visits, or hospital admissions) during the previous 2 weeks.

Current smoking and alcohol use were self‐reported (alcohol at least once a month). Physical activity was classified using the International Physical Activity Questionnaire (IPAQ)–Short Form^24^: individuals performing <150 min of moderate‐intensity or <75 min of vigorous‐intensity activity per week were classified as having insufficient physical activity.^25^ Cognitive performance was included as *z*‐scores to account for dementia severity as a predictor of mortality.

Functional status was measured through the number of basic Activities of Daily Living (ADLs) for which the participant required assistance. ADLs included walking across a room, dressing, bathing, eating, getting out of bed, toileting, and urinary and fecal continence. Each activity was rated on a 4‐point Likert scale: (1) no difficulty, (2) slight difficulty, (3) great difficulty but able to perform independently, and (4) unable to perform the task without assistance. Continence was rated on a separate 4‐point scale: (1) fully continent, (2) urinary incontinence, (3) fecal incontinence, and (4) dual incontinence. A global functionality score was created as a continuous measure comprising all ADL items.

### 2.3 Statistical analysis

All analyses accounted for the complex sampling design of ELSI‐Brazil using survey‐weighted procedures (svyset) in Stata/SE 17.0 (StataCorp, College Station, TX, USA). Person‐years of follow‐up were calculated for each participant from the date of the baseline interview (2015 to 2016) to either the date of death or the date of the second‐wave interview (2019 to 2021). Mortality rates were estimated for the full sample and stratified by dementia status, sex, and age group. Kaplan–Meier survival curves were generated for the overall sample and separately for men and women.

Cox proportional hazards regression models were fitted to estimate hazard ratios (HRs) and 95% confidence intervals (CIs). Four sequential models were specified with incremental adjustment. Model 1 included sociodemographic characteristics (age, sex, marital status, educational level, skin color, and urban vs rural residence). Model 2 added clinical and lifestyle variables (current smoking, current alcohol consumption, insufficient physical activity, recent use of health services, and number of chronic conditions). To examine the contribution of dementia severity to mortality, Model 3 further adjusted Model 2 for global cognitive performance, and Model 4 additionally included functional status. Sex interactions were tested for all variables that were statistically significant in Models 3 and 4.

#### 2.3.1 Sensitivity analysis

The cohort and sociodemographic characteristics of the final sample (*n* = 5249) and the 183 (3.4%) participants excluded (for whom dementia ascertainment was not possible) were described and compared using *t*‐test and chi‐squared.

### 2.4 Ethical considerations

The study was approved by the Research Ethics Committee of Fundação Oswaldo Cruz–Minas Gerais (protocol 34649814.3.0000.5091). This study was performed in accordance with the ethical standards as laid down in the 1964 Declaration of Helsinki and its later amendments. All participants provided written informed consent prior to participation. Data linkage procedures adhered to national ethical and confidentiality standards.

## 3 RESULTS

### 3.1 Participant characteristics

Of the 9412 participants in the ELSI‐Brazil baseline cohort, 5432 were aged ≥60 years. The final sample comprised 5249 participants, as it was not possible to ascertain dementia due to missing data in 183 individuals (3.4%). Among the 5249 included participants, 392 (6.4%) were classified as having dementia (364 individuals initially identified by the algorithm and 28 by a self‐reported previous diagnosis). The characteristics of the cohort by dementia status are presented in Table 1. Compared with those without dementia, participants with dementia were older at baseline (77.2 vs 69.5 years) and at death (81.4 vs 74.6 years), had a higher proportion of women (65.0% vs 54.9%), a lower proportion of individuals with a partner and who declared to be White, and generally had fewer years of formal education. Most participants in both groups resided in urban areas.

**TABLE 1:** General cohort characteristics according to dementia status (Brazil Longitudinal Study of Aging, 2015 to 2021).

	Dementia *n* = 392	Non dementia *n* = 4,857	Total *n* = 5,249
Cohort characteristics			
Total Person years of follow‐up	1,640	24,402.8	26,042.8
Years of follow‐up ‐mean (SD)	4.2 (1.8)	5.0 (1.0)	5.0(1.1)
Deaths: n (%)	153 (36.7%)	554 (10.8%)	707(12,5%)
Sociodemographic data			
Age at baseline‐ mean (SD)	77.2 (9.5)	69.5 (7.4)	70.1 (7.8)
Age at death‐ mean (SD)	81.4 (9.3)	74.6 (7.3)	75.1 (7.7)
Female gender Baseline: n (%)	266 (65.0)	2,878 (54.9)	3,144 (55.6)
Married/cohabiting: n(%)	129 (37.8)	2,594 (59.9)	2,723 (58.5)
Years of education (SD)	2.2 (3.1)	4.7 (4.1)	4.5 (4.1)
Skin color (non white) n(%)	250 (67.2)	2,681 (54.4)	2,931 (55.2)
Urban residence	303 (79.1)	4,113 (84,6)	4,416 (84.2)

### 3.2 Mortality rates

During 26,042.8 person‐years of follow‐up, 707 deaths occurred (12.5% of the cohort). Mortality was higher among individuals with dementia: 153 deaths (36.7%) yielded a mortality rate of 93.3 per 1000 person‐years (95% CI 79.6 to 109.3). Among those without dementia, 554 deaths (10.8%) resulted in a mortality rate of 22.7 per 1000 person‐years (95% CI 20.9 to 24.7).

**FIGURE 2:**
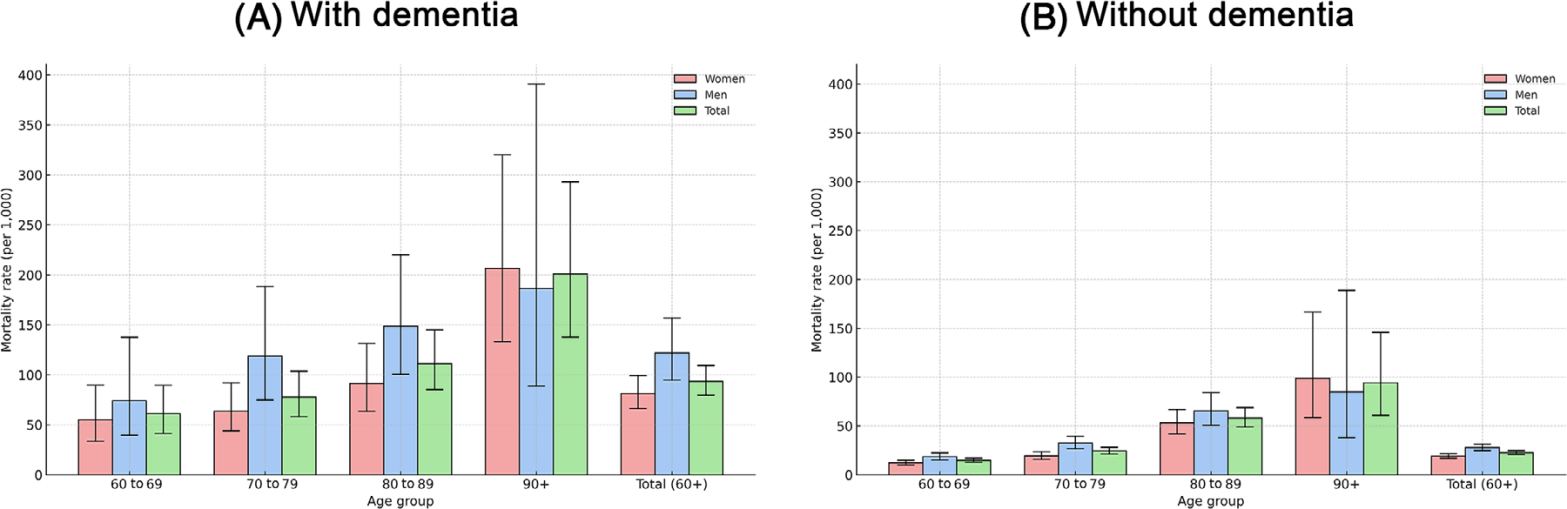
Mortality rates per 1000 person‐years among people with and without dementia stratified by sex and age groups. Graph depicting mortality rates by age and sex for participants with dementia (A) and without dementia (B).

Mortality was higher among men in both groups. Among participants with dementia, the mortality rate was 121.8 per 1000 person‐years (95% CI 94.6 to 156.9) for men and 81.1 per 1000 person‐years (95% CI 66.1 to 99.3) for women. In the non‐dementia group, mortality rates were 27.9 (95% CI 24.8 to 31.5) and 19.2 (95% CI 17.1 to 21.6) per 1000 person‐years for men and women, respectively. Age‐ and sex‐stratified estimates appear in Figure 2 and Table S1.

The crude HR for mortality among participants with dementia versus those without dementia was 4.11 (95% CI 3.32 to 5.08). Adjustment for age and sex yielded an HR of 2.65 (95% CI 2.08 to 3.39).

### 3.3 Survival analysis

**FIGURE 3:**
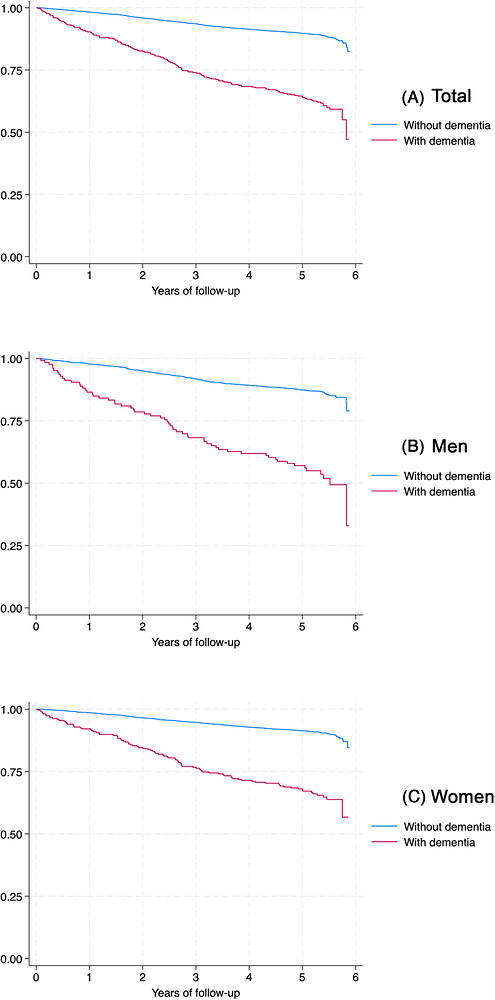
Kaplan–Meier survival curves according to dementia status for the whole sample and by sex (Brazil Longitudinal Study of Aging, 2015 to 2021). Graph depicting Kaplan–Meier survival curves for whole sample (A), men (B), and women (C).

Kaplan–Meier curves (Figure 3) demonstrated a clear and progressively widening separation between individuals with and without dementia. More than half of participants with dementia died during follow‐up, whereas survival among those without dementia remained above 80%. The divergence between curves appeared early and increased steadily over the 6‐year period.

Sex‐stratified curves showed similar patterns. Men had consistently lower survival than women overall, and the difference between dementia and non‐dementia groups was more pronounced among men. Women exhibited higher overall survival, though dementia was still associated with a marked reduction in survival.

### 3.4 Predictors of mortality

Among the 392 participants with dementia, complete data were available for 368 individuals in Model 1, 317 in Model 2, and 233 in Models 3 and 4. Full model results are shown in Table 2.

**TABLE 2:** Association between predictor variables and mortality risk among people with dementia (Brazil Longitudinal Study of Aging, 2015 to 2021).

Variable	Model 1 HR (95% CI)	Model 2 HR (95% CI)	Model 3 HR (95% CI)	Model 4 HR (95% CI)
**Sociodemographic characteristics**				
Age at baseline	**1.04 (1.02 to 1.07)**	**1.04 (1.01 to 1.07)**	1.00 (0.96 to 1.04)	1.00 (0.97 to 1.03)
Male gender	**1.58 (1.09 to 2.29)**	**1.94 (1.26 to 2.99)**	**2.46 (1.44 to 4.20)**	**2.01 (1.11 to 3.66)**
Married/cohabiting	0.68 (0.42 to 1.10)	**0.52 (0.31 to 0.88)**	**0.34 (0.17 to 0.67)** ^a^	**0.37 (0.19 to 0.73)** ^b^
Years of education	**1.07 (1.1 to 1.14)**	**1.07 (1.01 to 1.15)**	**1.18 (1.09 to 1.28)**	**1.21 (1.10 to 1.34)**
Skin color (non‐White)	1.09 (0.72 to 1.65)	1.15 (0.72 to 1.82)	0.80 (0.45 to 1.42)	0.95 (0.52 to 1.74)
Urban residence	0.90 (0.50 to 1.62)	0.80 (0.40 to 1.60)	0.98 (0.40 to 2.35)	0.85 (0.35 to 2.04)
**Health‐related variables**				
Current alcohol drinking	—	0.65 (0.25 to 1.67)	0.99 (0.38 to 2.59)	1.03 (0.36 to 2.94)
Current smoking	—	1.18 (0.57 to 2.46)	1.06 (0.43 to 2.60)	0.93 (0.41 to 2.14)
Insufficient physical activity	—	**2.40 (1.22 to 4.72)**	**2.13 (1.19 to 3.81)**	1.61 (0.82 to 3.15)
Number of chronic conditions	—	1.09 (0.95 to 1.26)	0.97 (0.79 to 1.19)	0.88 (0.72 to 1.08)
Access to any health services	—	0.96 (0.47 to 1.96)	1.05 (0.43 to 2.55)	0.96 (0.40 to 2.32)
**Dementia severity**				
Total cognitive score	—	—	**0.43 (0.20 to 0.92)**	**0.44 (0.21 to 0.96)**
Basic Activities of Daily Leaving	—	—	—	**1.13 (1.06 to 1.20)**

*Note*: Model 1: Mutually adjusted for: age, sex, marital status, educational level skin color, and place of residence. Model 2: Model 1 plus current tobacco and alcohol consumption, insufficient physical activity, access to health services, and number of chronic conditions. Model 3: Model 2 plus total cognitive score. Model 4: Model 3 plus total score on basic Activities of Daily Living.

Abbreviations: CI: confidence interval; HR, hazard ratio.

Bold values indicate statistical significance *p* < 0.05.

^a^ Sex interaction test (*p* = 0.031). Women (HR = 0.89; 95% CI: 0.40 to 1.96); men (HR = 0.19; 95% CI 0.04 to 0.99).

^b^ Sex interaction test (*p* = 0.037). Women (HR = 1.19; 95% CI: 0.58 to 2.44); men (HR = 0.20; 95% CI: 0.038 to 1.0).

#### 3.4.1 Sociodemographic factors

Baseline age was associated with increased mortality in Model 1 (HR = 1.04; 95% CI 1.02 to 1.07) and Model 2 (HR = 1.04; 95% CI 1.01 to 1.07), but this association was no longer significant after inclusion of cognitive performance (Model 3) and both cognition and functional status (Model 4).

Male sex was a consistent predictor of higher mortality across all models, with a fully adjusted HR of 2.01 (95% CI 1.11 to 3.66). Having a partner was associated with lower mortality risk in Models 2 to 4, and significant sex interactions (Model 3: *p* = 0.031; Model 4: *p* = 0.037) indicated that the protective effect was substantially stronger among men (HR = 0.19; 95% CI 0.04 to 0.99) than among women (HR = 0.89; 95% CI 0.40 to 1.96).

Higher educational attainment was associated with increased mortality risk in all models (HRs ranging from 1.07 to 1.21). Skin color and urban residence were not significantly associated with mortality risk.

#### 3.4.2 Health‐related factors and dementia severity

The number of chronic conditions, current smoking, alcohol consumption, and recent use of health services were not significantly associated with mortality in any model. Insufficient physical activity was associated with higher mortality in Model 2 (HR = 2.40; 95% CI 1.22 to 4.72) and Model 3 (HR = 2.13; 95% CI 1.19 to 3.81), but the association was no longer significant after adjusting for functional status in Model 4 (HR = 1.28; 95% CI 0.66 to 2.47).

Higher global cognitive performance was associated with lower mortality in Model 3 (HR = 0.43; 95% CI 0.20 to 0.92) and Model 4 (HR = 0.44; 95% CI 0.21 to 0.96). Conversely, poorer functional status was strongly associated with increased mortality in the fully adjusted model (HR = 1.13; 95% CI 1.06 to 1.21; *p* < 0.001).

#### 3.4.3 Sensitivity analysis

A comparison between the final sample of 5249 (96.4%) and the 183 (3.4%) participants for whom dementia ascertainment was not possible, and who were therefore not included in the final sample, showed that excluded individuals were significantly older (77.1 vs 70.1 years; *p* < 0.001), had fewer years of formal education (mean 1.7 vs 4.5 years; *p* < 0.001), were less likely to be married/cohabitating (35.0% vs 51.9%; *p* < 0.001), were less likely to live in urban areas (74.9% vs 80.1%; *p* = 0.001), and had a higher number of deaths during the follow‐up period (31.7% vs 13.5%; *p* < 0.001). Full comparisons are provided in Table S2.

## 4 DISCUSSION

This nationally representative study provides new evidence on dementia‐related mortality in Brazil, a large middle‐income country where population aging is rapidly accelerating.^2^ Individuals with dementia experienced markedly higher mortality than those without the condition, with a rate of 93.3 per 1000 person‐years – well within the range reported in other LMICs (59.5 to 216.1 per 1000 person‐years).^11^ The age‐ and sex‐adjusted HR of 2.65 was also consistent with estimates from the 10/66 Dementia Research Group in countries such as the Dominican Republic (2.22), Mexico (2.70), and India (2.33) and closely aligned with a meta‐analytic pooled estimate of 2.77 (95% CI 2.47 to 3.10).^11^ Notably, the estimate observed in the present study was lower than that found in the only prior Brazilian population‐based study conducted over 20 years ago (HR = 5.16),^17^ underscoring the importance of contemporary, nationally representative data to understand mortality dynamics in the country.

Several predictors of mortality emerged in this cohort. Male sex, lower cognitive performance, functional impairment, and insufficient physical activity were associated with higher mortality, in line with international evidence.^12^ Functional impairment was one of the strongest health‐related predictors of mortality, remaining significant even in the model adjusted for cognitive performance and sociodemographic factors. Once functional status was incorporated into the model, the association between insufficient physical activity and mortality was attenuated and no longer significant, suggesting that low physical activity may influence mortality primarily through its contribution to functional decline rather than operating as an independent risk factor. These findings are consistent with prior 10/66 results, which identified physical impairments – such as prior stroke or weakness – as important contributors to mortality in LMIC settings,^12^ supporting the role of physical frailty and dementia severity in shaping survival.

The sex differential observed – substantially higher mortality risk among men – is consistent with global patterns reported for both general and dementia‐specific mortality.^26^, ^27^ Several hypotheses have been proposed to explain this phenomenon, including differences in self‐care, risk‐taking behaviors, and hormonal changes that shape comorbidity profiles.^26^, ^28^ In the context of dementia, as men age, risk‐taking behaviors tend to decline, reducing their contribution to mortality differences, whereas hormonal influences on cardiovascular risk persist throughout later life.^28^ The protective effect of having a partner observed in this study is also consistent with prior literature, with the magnitude of this association generally stronger among men.^29^

Higher educational attainment was associated with increased mortality risk in all models, a striking finding that deserves careful consideration. Previous studies reported mixed findings, with some showing no association between education and mortality,^30^ others suggesting a protective effect,^31^ and still others – consistent with the present findings – indicating higher mortality among individuals with more years of schooling.^32^

While education is traditionally viewed as a protective factor against mortality in the general population – conferring benefits through healthier behaviors, higher socioeconomic status, and better access to healthcare – these paradoxical effects in dementia populations are not unheard of.^32^ One plausible explanation is the concept of cognitive reserve paradox: Individuals with higher education typically have greater cognitive reserve, allowing them to tolerate more extensive neuropathological damage before clinical symptoms become evident.^33^ As a result, they may receive a dementia diagnosis later in the disease trajectory, when the underlying pathology is already more advanced. This diagnostic delay could lead to a shorter interval between diagnosis and death, thereby increasing mortality risk once dementia is identified.^32^ This hypothesis is supported by neuroimaging studies showing that among individuals with similar clinical severity, those with higher education often exhibit a greater burden of Alzheimer's disease pathology (e.g., amyloid plaques and neurofibrillary tangles) at the time of diagnosis.^34^

Second, educational attainment in Brazil is closely intertwined with socioeconomic inequalities that may shape dementia outcomes in complex ways. Although higher education generally confers advantages, it may also be associated with lifestyle factors, such as alcohol use,^35^ that paradoxically increase vulnerability in late life.

It is also possible that this association reflects residual confounding by dementia severity or other unmeasured factors, despite our adjustments for cognitive performance and functional status. These findings underscore the complexity of the relationship between education and mortality in dementia and highlight the need for further research to disentangle the underlying mechanisms.

Baseline age, although initially associated with mortality, as reported in several previous studies,^8^, ^9^, ^10^, ^11^, ^12^ was no longer significant after adjustment for cognitive performance and functional status. This pattern supports the hypothesis that disease severity, rather than chronological age, plays a dominant role in survival among individuals with dementia.

In our sensitivity analysis, we found that individuals excluded from our analysis (*n* = 183) were older, lived alone, lived in rural areas, and had less education and higher mortality. These excluded participants were probably too ill to participate in cognitive testing (which could be due to dementia or other health conditions) or did not engage due to refusal/low literacy and did not have a family member to answer the IQCODE. This pattern suggests that the excluded group may have had a higher probability of dementia, potentially leading to a slight underestimation of dementia prevalence and dementia‐related mortality rates.

This study has important strengths, including the use of a large, nationally representative cohort that allows for a robust assessment of mortality risk factors generalizable to the Brazilian older population. The inclusion of multiple covariates – sociodemographic, clinical, cognitive, and functional – provides a comprehensive analysis of how different factors influence mortality among individuals with dementia. Additionally, the linkage with Brazil's national SIM strengthens the validity of the results. However, some limitations must be acknowledged. First, the ELSI‐Brazil study was not initially designed to diagnose dementia, and the published algorithm used to classify dementia cases may not perfectly reflect clinical diagnoses. This limitation could lead to misclassification bias, potentially resulting in either underestimation or overestimation of mortality risk. Furthermore, in a country as large and diverse as Brazil, the interpretation of findings may not be universal, given regional disparities in factors such as healthcare access.

Despite these considerations, the findings have important implications for public health and dementia care in Brazil and other LMICs. The high mortality rate associated with dementia highlights the urgency of improving early detection, expanding access to care, and implementing interventions aimed at slowing functional decline. The strong influence of social support – reflected in the protective effect of having a partner – underscores the need for community‐based care models that integrate family and caregiver support. Promoting physical activity in older adults, particularly those with early cognitive impairment, may also help preserve function and improve survival outcomes. The association between higher education and mortality reinforces the need for diagnostic strategies sensitive to cognitive reserve and culturally appropriate for diverse populations. Collectively, these findings contribute to a deeper understanding of dementia‐related mortality in LMIC settings and emphasize the importance of targeted, context‐specific strategies to improve outcomes for people living with dementia.

## AUTHOR CONTRIBUTIONS

We confirm that all listed authors have contributed to this manuscript and that every significant contributor has been included. Matheus Ghossain Barbosa is the main author and led the conceptualization, data analysis, writing, reviewing and submitting processes. Juliana Vaz de Melo Mambrini was responsible for the linkage with the mortality national system and contributed to the writing and reviewing processes. Wendell Lima Rabelo contributed to the conceptualization, writing, and reviewing processes. Andrew Cristopher Claro Miguel contributed to the writing and reviewing processes. Lucas Teixeira contributed to the writing, reviewing, and submitting. Maria Fernanda Lima‐Costa led the cohort design and data collection and contributed to writing and reviewing. Cleusa P. Ferri supervised and contributed to conceptualization, data analysis, writing, reviewing, and submitting. All authors have read and approved the manuscript.

## CONFLICT OF INTEREST STATEMENT

The authors report no conflicts of interest. All author disclosures are available in the Supporting Information.

## Consent Statement

All participants in this study provided written informed consent prior to participation.

## Supporting information



Supporting Information

Supporting Information

Supporting Information

## References

[1] Institute for Health Metrics and Evaluation (IHME) . GBD Compare. IHME, University of Washington; 2025. Accessed October 28, 2025. Available at: https://vizhub.healthdata.org/gbd‐compare/

[2] GBD 2019 Dementia Forecasting Collaborators . Estimation of the global prevalence of dementia in 2019 and forecasted prevalence in 2050: an analysis for the Global Burden of Disease Study 2019. Lancet Public Health. 2022;7(2):e105‐e125. doi:10.1016/S2468-2667(21)00249-8 34998485 PMC8810394

[3] Brunnström HR , Englund EM . Cause of death in patients with dementia disorders. Eur J Neurol. 2009;16(4):488‐492. 19170740 10.1111/j.1468-1331.2008.02503.x

[4] Alzheimer's disease international. Numbers of people with dementia worldwide: an update to the estimates. Alzheimer's Disease International; 2020. Accessed October 28, 2025. Available at: https://www.alzint.org/resource/numbers‐of‐people‐with‐dementia‐worldwide

[5] Aliberti MJR , Suemoto CK , Laks J , et al. Mapping the numbers of dementia in Brazil: a Delphi consensus study. Int J Geriatr Psychiatry. 2025;40(2):e70055. 39938925 10.1002/gps.70055

[6] da Mata FAF , Ramos AA , Bertola L , Suarez T , Ferri CP , de Oliveira Júnior HA . The cost of dementia in Brazil. Braz J Psychiatry. 2025;47:e20243611. 39550769 10.47626/1516-4446-2024-3611 PMC12681328

[7] Todd S , Barr S , Roberts M , Passmore AP . Survival in dementia and predictors of mortality: a review. Int J Geriatr Psychiatry. 2013;28(11):1109‐1124. 23526458 10.1002/gps.3946

[8] Ono R , Sakurai T , Sugimoto T , et al. Mortality risks and causes of death by dementia types in a Japanese cohort with dementia: nCGG‐STORIES. J Alzheimers Dis. 2023;92(2):487‐498. 36776074 10.3233/JAD-221290 PMC10041427

[9] González PTM , Vieira LM , Sarmiento APY , Ríos JS , Alarcón MAS , Guerrero MAO . Predictors of mortality in dementia: a systematic review and meta‐analysis. Neurol Perspect. 2024;4(4):100175.

[10] Mobaderi T , Kazemnejad A , Salehi M . Exploring the impacts of risk factors on mortality patterns of global Alzheimer's disease and related dementias from 1990 to 2021. Sci Rep. 2024;14(1):15583. 38971870 10.1038/s41598-024-65887-4 PMC11227499

[11] Brodaty H , Seeher K , Gibson L . Dementia time to death: a systematic literature review on survival time and years of life lost in people with dementia. Int Psychogeriatr. 2012;24(7):1034‐1045. 22325331 10.1017/S1041610211002924

[12] Prince M , Acosta D , Ferri CP , et al. Dementia incidence and mortality in middle‐income countries, and associations with indicators of cognitive reserve: a 10/66 Dementia Research Group population‐based cohort study. Lancet Lond Engl. 2012;380(9836):50‐58. 10.1016/S0140-6736(12)60399-7 PMC3525981 22626851

[13] Piovezan RD , Oliveira D , Arias N , Acosta D , Prince MJ , Ferri CP . Mortality rates and mortality risk factors in older adults with dementia from low‐ and middle‐income countries: the 10/66 Dementia Research Group population‐based cohort study. J Alzheimers Dis. 2020;75(2):581‐593. 32310178 10.3233/JAD-200078 PMC7306886

[14] Lopes MA , Nassar SM , Barcelos‐Ferreira R , Folquitto JC , Litvoc J . Incidence of dementia in a population cohort of older people from São Paulo, Brazil. Int J Geriatr Psychiatry. 2021;1‐10. 10.1002/gps.5660 34802177

[15] César‐Freitas KG , Suemoto CK , Power MC , Brucki SMD , Nitrini R . Incidence of dementia in a Brazilian population: the Tremembé Epidemiologic Study. Alzheimers Dement J Alzheimers Assoc. 2022;18(4):581‐590. 10.1002/alz.12423 34338427

[16] Nitrini R , Caramelli P , Herrera E , et al. Incidence of dementia in a community‐dwelling Brazilian population. Alzheimer Dis Assoc Disord. 2004;18(4):241‐246. 15592138

[17] Nitrini R , Caramelli P , Herrera E , et al. Mortality from dementia in a community‐dwelling Brazilian population. Int J Geriatr Psychiatry. 2005;20(3):247‐253. 15717343 10.1002/gps.1274

[18] Sonnega A , Faul JD , Ofstedal MB , Langa KM , Phillips JW , Weir DR . Cohort profile: the Health and Retirement Study (HRS). Int J Epidemiol. 2014;43(2):576‐585. doi:10.1093/ije/dyu067. Epub 2014 Mar 25. PMID: 24671021; PMCID: PMC3997380. 24671021 PMC3997380

[19] Lima‐Costa MF , de Andrade FB , de Souza PRB , et al. The Brazilian Longitudinal Study of Aging (ELSI‐Brazil): objectives and design. Am J Epidemiol. 2018;187(7):1345‐1353. 29394304 10.1093/aje/kwx387 PMC6031009

[20] Lima‐Costa MF , de Melo Mambrini JV , Bof de Andrade F , et al. Cohort profile: the Brazilian Longitudinal Study of Ageing (ELSI‐Brazil). Int J Epidemiol. 2023;52(1):e57‐e65. doi:10.1093/ije/dyac132 35748356 PMC9908056

[21] Bertola L , Suemoto CK , Aliberti MJR , et al. Prevalence of dementia and cognitive impairment no dementia in a large and diverse nationally representative sample: the ELSI‐Brazil Study. J Gerontol A Biol Sci Med Sci. 2023;78(6):1060‐1068. 36682021 10.1093/gerona/glad025

[22] Dang L , Dong L , Mezuk B . Shades of blue and gray: a comparison of the Center for Epidemiologic Studies Depression Scale and the Composite International Diagnostic Interview for assessment of depression syndrome in later life. Gerontologist. 2020;60(4):e242‐e253.22. 31112598 10.1093/geront/gnz044 PMC7228460

[23] National Institute on Alcohol Abuse and Alcoholism (NIAAA) . Understanding alcohol drinking patterns. NIAAA; 2025. [date unknown]. Accessed November 8, 2025.23. Available at: https://www.niaaa.nih.gov/

[24] Lee PH , Macfarlane DJ , Lam T , Stewart SM . Validity of the international physical activity questionnaire short form (IPAQ‐SF): a systematic review. Int J Behav Nutr Phys Act. 2011;8(1):115. 22018588 10.1186/1479-5868-8-115 PMC3214824

[25] Bull FC , Al‐Ansari SS , Biddle S , et al. World Health Organization 2020 guidelines on physical activity and sedentary behaviour. Br J Sports Med. 2020;54(24):1451‐1462. 33239350 10.1136/bjsports-2020-102955 PMC7719906

[26] Crimmins EM , Shim H , Zhang YS , Kim JK . Differences between men and women in mortality and the health dimensions of the morbidity process. Clin Chem. 2019;65(1):135‐145. 30478135 10.1373/clinchem.2018.288332 PMC6345642

[27] Gambassi G , Lapane KL , Landi F , Sgadari A , Mor V , Bernabie R . Gender differences in the relation between comorbidity and mortality of patients with Alzheimer's disease. Systematic Assessment of Geriatric drug use via Epidemiology (SAGE) Study Group. Neurology. 1999;53(3):508‐516. 10449112 10.1212/wnl.53.3.508

[28] Zhao E , Crimmins EM . Mortality and morbidity in ageing men: biology, lifestyle and environment. Rev Endocr Metab Disord. 2022;23(6):1285‐1304. 35697963 10.1007/s11154-022-09737-6 PMC9748037

[29] Robards J , Evandrou M , Falkingham J , Vlachantoni A . Marital status, health and mortality. Maturitas. 2012;73(4):295‐299. 23007006 10.1016/j.maturitas.2012.08.007 PMC3635122

[30] Llinàs‐Regla J , López‐Pousa S , Vilalta‐Franch J , Garre‐Olmo J , Román GC . Mortality after a diagnosis of dementia in a population aged 75 and over in Spain. Neuroepidemiology. 2008;31(2):80‐88. 18622143 10.1159/000144088

[31] Agüero‐Torres H , Fratiglioni L , Guo Z , Viitanen M , Winblad B . Mortality from dementia in advanced age: a 5‐year follow‐up study of incident dementia cases. J Clin Epidemiol. 1999;52(8):737‐743. 10465318 10.1016/s0895-4356(99)00067-0

[32] Stern Y , Tang MX , Denaro J , Mayeux R . Increased risk of mortality in Alzheimer's disease patients with more advanced educational and occupational attainment. Ann Neurol. 1995;37(5):590‐595. 7755353 10.1002/ana.410370508

[33] Stern Y . Cognitive reserve in ageing and Alzheimer's disease. Lancet Neurol. 2012;11(11):1006‐1012. 23079557 10.1016/S1474-4422(12)70191-6 PMC3507991

[34] Bennett DA , Wilson RS , Schneider JA , et al. Education modifies the relation of AD pathology to level of cognitive function in older persons. Neurology. 2003;60(12):1909‐1915. 12821732 10.1212/01.wnl.0000069923.64550.9f

[35] Plens JA , Valente JY , Mari JJ , Ferrari G , Sanchez ZM , Rezende LFM . Patterns of alcohol consumption in Brazilian adults. Sci Rep. 2022;12(1):8603. 35597775 10.1038/s41598-022-12127-2 PMC9123624

