# Supplementary material for: Dementia and all‐cause mortality in older adults: Findings from the ELSI‐Brazil study
Source: Alzheimers Dement. 2026 May 6;22(5):e71400. doi: 10.1002/alz.71400 (PMC13149212; doi:10.1002/alz.71400)
Supplement: Supplementary file 1 — Supporting Information [file ALZ-22-e71400-s003.pdf]

ICMJE DISCLOSURE FORM

**Date:**	March 1st, 2026
**Your Name:**	Matheus Ghossain Barbosa
**Manuscript Title:**	Dementia and All-Cause Mortality in Older Adults: Findings from the ELSI-Brazil Study
**Manuscript Number (if known):**	ADJ-D-25-03330

In the interest of transparency, we ask you to disclose all relationships/activities/interests listed below that are related to the content of your manuscript. "Related" means any relation with for-profit or not-for-profit third parties whose interests may be affected by the content of the manuscript. Disclosure represents a commitment to transparency and does not necessarily indicat a bias. If you are in doubt about whether to list a relationship/activity/interest, it is preferable that you do so.

The author's relationships/activities/interests should be defined broadly. For example, if your manuscript pertains to the epidemiology of hypertension, you should declare all relationships with manufacturers of antihypertensive medication, even if tha medication is not mentioned in the manuscript.

In item #1 below, report all support for the work reported in this manuscript without time limit. For all other items, the time fram for disclosure is the past 36 months.

		**Name all entities with whom you have this relationship or indicate none (add rows as needed)**	**Specifications/Comments (e.g., if payments were made to you or to your institution)**
**Time frame: Since the initial planning of the work**			
**1**	All support for the present manuscript (e.g., funding, provision of study materials, medical writing, article processing charges, etc.)<br>**No time limit for this item.**	**None**	
**Time frame: past 36 months**			
**2**	Grants or contracts from any entity (if not indicated in item #1 above).	**None**	
**3**	Royalties or licenses	**None**	

		Name all entities with whom you have this relationship or indicate none (add rows as [illegible]	Specifications/Comments (e.g., if payments were made to you or to your institution)
**4**	Consulting fees	**None**	
**5**	Payment or honoraria for lectures, presentations, speakers bureaus, manuscript writing or educational events	Johnson & Johnson<br>M8 Pharma<br>Libbs	
**6**	Payment for expert testimony	**None**	
**7**	Support for attending meetings and/or travel	Johnson & Johnson<br>M8 Pharma<br>Daiichi Sankyo<br>Lundbeck	
**8**	Patents planned, issued or pending	**None**	
**9**	Participation on a Data Safety Monitoring Board or Advisory Board	**None**	

		Name all entities with whom you have this relationship or indicate none (add rows as needed)	Specifications/Comments (e.g., if payments were made to you or to your institution)
**10**	Leadership or fiduciary role in other board, society, committee or advocacy group, paid or unpaid	**None**	
**11**	Stock or stock options	**None**	
**12**	Receipt of equipment, materials, drugs, medical writing, gifts or other services	**None**	
**13**	Other financial or non-financial interests	**None**	

Please place an "X" next to the following statement to indicate your agreement:

X I certify that I have answered every question and have not altered the wording of any of the questions on this form.

ICMJE DISCLOSURE FORM

**Date:**	2/21/2026
**Your Name:**	Andrew Christopher Claro Miguel
**Manuscript Title:**	Dementia and All-Cause Mortality in Older Adults: Findings from the ELSI-Brazil Study
**Manuscript Number (if known):**	ADJ-D-25-03330

In the interest of transparency, we ask you to disclose all relationships/activities/interests listed below that are related to the content of your manuscript. “Related” means any relation with for-profit or not-for-profit third parties whose interests may be affected by the content of the manuscript. Disclosure represents a commitment to transparency and does not necessarily indicat a bias. If you are in doubt about whether to list a relationship/activity/interest, it is preferable that you do so.

The author’s relationships/activities/interests should be defined broadly. For example, if your manuscript pertains to the epidemiology of hypertension, you should declare all relationships with manufacturers of antihypertensive medication, even if tha medication is not mentioned in the manuscript.

In item #1 below, report all support for the work reported in this manuscript without time limit. For all other items, the time fram for disclosure is the past 36 months.

		Name all entities with whom you have this relationship or indicate none (add rows as needed)	Specifications/Comments (e.g., if payments were made to you or to your institution)
**Time frame: Since the initial planning of the work**			
**1**	All support for the present manuscript (e.g., funding, provision of study materials, medical writing, article processing charges, etc.)<br>**No time limit for this item.**	☒ **None**<br><br>Click the tab key to add additional rows.	
**Time frame: past 36 months**			
**2**	Grants or contracts from any entity (if not indicated in item #1 above).	☒ **None**	
**3**	Royalties or licenses	☒ **None**	
**4**	Consulting fees	☒ **None**	

		Name all entities with whom you have this relationship or indicate none (add rows as needed)	Specifications/Comments (e.g., if payments were made to you or to your institution)
**5**	Payment or honoraria for lectures, presentations, speakers bureaus, manuscript writing or educational events	☒ **None**	
**6**	Payment for expert testimony	☒ **None**	
**7**	Support for attending meetings and/or travel	☐ **None**<br>Johnson & Johnson]	Registration for congresses and courses – to me
**8**	Patents planned, issued or pending	☒ **None**	
**9**	Participation on a Data Safety Monitoring Board or Advisory Board	☒ **None**	
**10**	Leadership or fiduciary role in other board, society, committee or advocacy group, paid or unpaid	☒ **None**	
**11**	Stock or stock options	☐ **None**<br>Eli Lilly<br>Novo-Nordisk	 Personal stock investment in the US<br>Personal stock investment in the US

		Name all entities with whom you have this relationship or indicate none (add rows as needed)		Specifications/Comments (e.g., if payments were made to you or to your institution)
12	Receipt of equipment, materials, drugs, medical writing, gifts or other services	☐	**None**	
		Libbs	Gifts and materials – to me	
13	Other financial or non-financial interests	☒	**None**	

Please place an "X" next to the following statement to indicate your agreement:

☒ I certify that I have answered every question and have not altered the wording of any of the questions on this form.

ICMJE DISCLOSURE FORM

**Date:**	March 9, 2026
**Your Name:**	Cleusa Pinheiro Ferri
**Manuscript Title:**	Dementia and All-Cause Mortality in Older Adults: Findings from the ELSI-Brazil Study
**Manuscript Number (if known):**	ADJ-D-25-03330

In the interest of transparency, we ask you to disclose all relationships/activities/interests listed below that are related to the content of your manuscript. "Related" means any relation with for-profit or not-for-profit third parties whose interests may be affected by the content of the manuscript. Disclosure represents a commitment to transparency and does not necessarily indicat a bias. If you are in doubt about whether to list a relationship/activity/interest, it is preferable that you do so.

The author's relationships/activities/interests should be defined broadly. For example, if your manuscript pertains to the epidemiology of hypertension, you should declare all relationships with manufacturers of antihypertensive medication, even if th medication is not mentioned in the manuscript.

In item #1 below, report all support for the work reported in this manuscript without time limit. For all other items, the time fram for disclosure is the past 36 months.

	Name all entities with whom you have this relationship or indicate none (add rows as needed)	Specifications/Comments (e.g., if payments were made to you or to your institution)
**Time frame: Since the initial planning of the work**		

		Name all entities with whom you have this relationship or indicate none (add rows as [illegible]	Specifications/Comments (e.g., if payments were made to you or to your institution)
**1**	All support for the present manuscript (e.g., funding, provision of study materials, medical writing, article processing charges, etc.)<br>**No time limit for this item.**	**None**	
**Time frame: past 36 months**			
**2**	Grants or contracts from any entity (if not indicated in item #1 above).	**None**	
**3**	Royalties or licenses	**None**	
**4**	Consulting fees	**None**	
**5**	Payment or honoraria for lectures, presentations, speakers bureaus, manuscript writing or educational events	**None**	
**6**	Payment for expert testimony	**None**	

		**Name all entities with whom you have this relationship or indicate none (add rows as needed)**	**Specifications/Comments (e.g., if payments were made to you or to your institution)**
**7**	Support for attending meetings and/or travel	**None**	
**8**	Patents planned, issued or pending	**None**	
**9**	Participation on a Data Safety Monitoring Board or Advisory Board	**None**	
**10**	Leadership or fiduciary role in other board, society, committee or advocacy group, paid or unpaid	**None**	
**11**	Stock or stock options	**None**	
**12**	Receipt of equipment, materials, drugs, medical writing, gifts or other services	**None**	
**13**	Other financial or non-financial interests	**None**	

	Name all entities with whom you have this relationship or indicate none (add rows as needed)	Specifications/Comments (e.g., if payments were made to you or to your institution)

Please place an "X" next to the following statement to indicate your agreement:

X I certify that I have answered every question and have not altered the wording of any of the questions on this form.

ICMJE DISCLOSURE FORM

**Date:**	March 9, 2026
**Your Name:**	Maria Fernanda Lima-Costa
**Manuscript Title:**	Dementia and All-Cause Mortality in Older Adults: Findings from the ELSI-Brazil Study
**Manuscript Number (if known):**	ADJ-D-25-03330

In the interest of transparency, we ask you to disclose all relationships/activities/interests listed below that are related to the content of your manuscript. "Related" means any relation with for-profit or not-for-profit third parties whose interests may be affected by the content of the manuscript. Disclosure represents a commitment to transparency and does not necessarily indicat[illegible] a bias. If you are in doubt about whether to list a relationship/activity/interest, it is preferable that you do so.

The author's relationships/activities/interests should be defined broadly. For example, if your manuscript pertains to the epidemiology of hypertension, you should declare all relationships with manufacturers of antihypertensive medication, even if th[illegible] medication is not mentioned in the manuscript.

In item #1 below, report all support for the work reported in this manuscript without time limit. For all other items, the time fram[illegible] for disclosure is the past 36 months.

		Name all entities with whom you have this relationship or indicate none (add rows as needed)	Specifications/Comments (e.g., if payments were made to you or to your institution)
**Time frame: Since the initial planning of the work**			
**1**	All support for the present manuscript (e.g., funding, provision of study materials, medical writing, article processing charges, etc.)<br>**No time limit for this item.**	**None**	
**Time frame: past 36 months**			
**2**	Grants or contracts from any entity (if not indicated in item #1 above).	**None**	

		Name all entities with whom you have this relationship or indicate none (add rows as [illegible]	Specifications/Comments (e.g., if payments were made to you or to your institution)
**3**	Royalties or licenses	**None**	
**4**	Consulting fees	**None**	
**5**	Payment or honoraria for lectures, presentations, speakers bureaus, manuscript writing or educational events	**None**	
**6**	Payment for expert testimony	**None**	
**7**	Support for attending meetings and/or travel	**None**	
**8**	Patents planned, issued or pending	**None**	

		Name all entities with whom you have this relationship or indicate none (add rows as needed)	Specifications/Comments (e.g., if payments were made to you or to your institution)
**9**	Participation on a Data Safety Monitoring Board or Advisory Board	**None**	
**10**	Leadership or fiduciary role in other board, society, committee or advocacy group, paid or unpaid	**None**	
**11**	Stock or stock options	**None**	
**12**	Receipt of equipment, materials, drugs, medical writing, gifts or other services	**None**	
**13**	Other financial or non-financial interests	**None**	

Please place an “X” next to the following statement to indicate your agreement:

X I certify that I have answered every question and have not altered the wording of any of the questions on this form.

ICMJE DISCLOSURE FORM

**Date:**	March 9, 2026
**Your Name:**	Juliana Vaz de Melo Mambrini
**Manuscript Title:**	Dementia and All-Cause Mortality in Older Adults: Findings from the ELSI-Brazil Study
**Manuscript Number (if known):**	ADJ-D-25-03330

In the interest of transparency, we ask you to disclose all relationships/activities/interests listed below that are related to the content of your manuscript. "Related" means any relation with for-profit or not-for-profit third parties whose interests may be affected by the content of the manuscript. Disclosure represents a commitment to transparency and does not necessarily indicat a bias. If you are in doubt about whether to list a relationship/activity/interest, it is preferable that you do so.

The author's relationships/activities/interests should be defined broadly. For example, if your manuscript pertains to the epidemiology of hypertension, you should declare all relationships with manufacturers of antihypertensive medication, even if tha medication is not mentioned in the manuscript.

In item #1 below, report all support for the work reported in this manuscript without time limit. For all other items, the time fram for disclosure is the past 36 months.

		Name all entities with whom you have this relationship or indicate none (add rows as needed)	Specifications/Comments (e.g., if payments were made to you or to your institution)
**Time frame: Since the initial planning of the work**			
**1**	All support for the present manuscript (e.g., funding, provision of study materials, medical writing, article processing charges, etc.) **No time limit for this item.**	**None**	
**Time frame: past 36 months**			
**2**	Grants or contracts from any entity (if not indicated in item #1 above).	**None**	
**3**	Royalties or licenses	**None**	
**4**	Consulting fees	**None**	

		Name all entities with whom you have this relationship or indicate none (add rows as [illegible]	Specifications/Comments (e.g., if payments were made to you or to your institution)
**5**	Payment or honoraria for lectures, presentations, speakers bureaus, manuscript writing or educational events	**None**	
**6**	Payment for expert testimony	**None**	
**7**	Support for attending meetings and/or travel	**None**	
**8**	Patents planned, issued or pending	**None**	
**9**	Participation on a Data Safety Monitoring Board or Advisory Board	**None**	
**10**	Leadership or fiduciary role in other board, society, committee or advocacy group, paid or unpaid	**None**	

		Name all entities with whom you have this relationship or indicate none (add rows as [illegible])	Specifications/Comments (e.g., if payments were made to you or to your institution)
**11**	Stock or stock options	**None**	
**12**	Receipt of equipment, materials, drugs, medical writing, gifts or other services	**None**	
**13**	Other financial or non-financial interests	**None**	

Please place an "X" next to the following statement to indicate your agreement:

X I certify that I have answered every question and have not altered the wording of any of the questions on this form.

ICMJE DISCLOSURE FORM

**Date:**	March 9, 2026
**Your Name:**	Wendell Lima Rabelo
**Manuscript Title:**	Dementia and All-Cause Mortality in Older Adults: Findings from the ELSI-Brazil Study
**Manuscript Number (if known):**	ADJ-D-25-03330

In the interest of transparency, we ask you to disclose all relationships/activities/interests listed below that are related to the content of your manuscript. "Related" means any relation with for-profit or not-for-profit third parties whose interests may be affected by the content of the manuscript. Disclosure represents a commitment to transparency and does not necessarily indicat[illegible] a bias. If you are in doubt about whether to list a relationship/activity/interest, it is preferable that you do so.

The author's relationships/activities/interests should be defined broadly. For example, if your manuscript pertains to the epidemiology of hypertension, you should declare all relationships with manufacturers of antihypertensive medication, even if th[illegible] medication is not mentioned in the manuscript.

In item #1 below, report all support for the work reported in this manuscript without time limit. For all other items, the time fram[illegible] for disclosure is the past 36 months.

		Name all entities with whom you have this relationship or indicate none (add rows as needed)	Specifications/Comments (e.g., if payments were made to you or to your institution)
**Time frame: Since the initial planning of the work**			
**1**	All support for the present manuscript (e.g., funding, provision of study materials, medical writing, article processing charges, etc.)<br>**No time limit for this item.**	**None**	
**Time frame: past 36 months**			
**2**	Grants or contracts from any entity (if not indicated in item #1 above).	**None**	
**3**	Royalties or licenses	**None**	
**4**	Consulting fees	**None**	
**5**	Payment or honoraria for lectures, presentations, speakers bureaus, manuscript writing or educational events	**None**	
**6**	Payment for expert testimony	**None**	

		Name all entities with whom you have this relationship or indicate none (add rows as needed)	Specifications/Comments (e.g., if payments were made to you or to your institution)
**7**	Support for attending meetings and/or travel	**None**	
**8**	Patents planned, issued or pending	**None**	
**9**	Participation on a Data Safety Monitoring Board or Advisory Board	**None**	
**10**	Leadership or fiduciary role in other board, society, committee or advocacy group, paid or unpaid	**None**	
**11**	Stock or stock options	**None**	
**12**	Receipt of equipment, materials, drugs, medical writing, gifts or other services	**None**	
**13**	Other financial or non-financial interests	**None**	

	Name all entities with whom you have this relationship or indicate none (add rows as needed)	Specifications/Comments (e.g., if payments were made to you or to your institution)

Please place an "X" next to the following statement to indicate your agreement:

X I certify that I have answered every question and have not altered the wording of any of the questions on this form.

ICMJE DISCLOSURE FORM

**Date:**	March 9, 2026
**Your Name:**	Lucas Martins Teixeira
**Manuscript Title:**	Dementia and All-Cause Mortality in Older Adults: Findings from the ELSI-Brazil Study
**Manuscript Number (if known):**	ADJ-D-25-03330

In the interest of transparency, we ask you to disclose all relationships/activities/interests listed below that are related to the content of your manuscript. "Related" means any relation with for-profit or not-for-profit third parties whose interests may be affected by the content of the manuscript. Disclosure represents a commitment to transparency and does not necessarily indicat[illegible] a bias. If you are in doubt about whether to list a relationship/activity/interest, it is preferable that you do so.

The author's relationships/activities/interests should be defined broadly. For example, if your manuscript pertains to the epidemiology of hypertension, you should declare all relationships with manufacturers of antihypertensive medication, even if th[illegible] medication is not mentioned in the manuscript.

In item #1 below, report all support for the work reported in this manuscript without time limit. For all other items, the time fram[illegible] for disclosure is the past 36 months.

		Name all entities with whom you have this relationship or indicate none (add rows as needed)	Specifications/Comments (e.g., if payments were made to you or to your institution)
**Time frame: Since the initial planning of the work**			
**1**	All support for the present manuscript (e.g., funding, provision of study materials, medical writing, article processing charges, etc.) **No time limit for this item.**	**None**	
**Time frame: past 36 months**			
**2**	Grants or contracts from any entity (if not indicated in item #1 above).	**None**	

		Name all entities with whom you have this relationship or indicate none (add rows as needed)	Specifications/Comments (e.g., if payments were made to you or to your institution)
**3**	Royalties or licenses	**None**	
**4**	Consulting fees	**None**	
**5**	Payment or honoraria for lectures, presentations, speakers bureaus, manuscript writing or educational events	**None**	
**6**	Payment for expert testimony	**None**	
**7**	Support for attending meetings and/or travel	**None**	
**8**	Patents planned, issued or pending	**None**	

		Name all entities with whom you have this relationship or indicate none (add rows as needed)	Specifications/Comments (e.g., if payments were made to you or to your institution)
**9**	Participation on a Data Safety Monitoring Board or Advisory Board	**None**	
**10**	Leadership or fiduciary role in other board, society, committee or advocacy group, paid or unpaid	**None**	
**11**	Stock or stock options	**None**	
**12**	Receipt of equipment, materials, drugs, medical writing, gifts or other services	**None**	
**13**	Other financial or non-financial interests	**None**	

Please place an "X" next to the following statement to indicate your agreement:

X I certify that I have answered every question and have not altered the wording of any of the questions on this form.
