# Supplementary material for: Dementia and all‐cause mortality in older adults: Findings from the ELSI‐Brazil study
Source: Alzheimers Dement. 2026 May 6;22(5):e71400. doi: 10.1002/alz.71400 (PMC13149212; doi:10.1002/alz.71400)
Supplement: Supplementary file 2 — Supporting Information [file ALZ-22-e71400-s002.docx]

**Table 1S. Mortality rates per 1000 person-years among people with and without dementia stratified by sex and age (ELSI-Brasil, 2015-2021)**

|  | **Dementia**  Rate (95% CI) | | | **Non dementia**  Rate (95% CI) | | |
| --- | --- | --- | --- | --- | --- | --- |
| **Age group** (years) | **Female** n=266 | **Male**  n=126 | **Total**  n=392 | **Female**  n=2,878 | **Male**  n=1,979 | **Total** n=4,857 |
| **60-69** | 54.9 (33.6-89.6) | 74.0 (39.8-137.6) | 61.0 (41.5-89.5) | 12.3 (10.1-14.9) | 18.6 (15.3-22.5) | 14,8 (12.9-17.0) |
| **70-79** | 63.5 (43.8-91.9) | 118.7 (74.8-188.4) | 77.6 (58.1-103.6) | 19.4 (15.8-23.7) | 32.6 (26.8-39.6) | 24.5 (21.3-28.2) |
| **80-89** | 91.2 (63.4-131.3) | 148.6 (100.4-219.9) | 111.1 (85.1-145.0) | 53.0 (42.0-66.7) | 65.2 (50.6-84.0) | 57.9 (48.8-68.7) |
| **90+** | 206.5 (133.2-320.1) | 186.3 (88.8-390.8) | 200.8 (137.7-292.9) | 98.7 (58.5-166.6) | 84.8 (38.1-188.8) | 94.1 (60.7-145.8) |
| **Total (60+)** | 81.1 (66.1-99.3) | 121.8 (94.6-156.9) | 93.3 (79.6-109.3) | 19.2 (17.1-21.6) | 27.9 (24.8-31.5) | 22.7 (20.9-24.7) |
