# Supplementary material for: Dementia and all‐cause mortality in older adults: Findings from the ELSI‐Brazil study
Source: Alzheimers Dement. 2026 May 6;22(5):e71400. doi: 10.1002/alz.71400 (PMC13149212; doi:10.1002/alz.71400)
Supplement: Supplementary file 3 — Supporting Information [file ALZ-22-e71400-s001.docx]

**Table S1. Cohort characteristics according to baseline missing data**

|  | **Excluded due to missing data**  **n=183**  **n(%)** | **Included**  **n=5,249**  **n(%)** | **Total**  **n=5,432**  **n(%)** | **p-value**  **t-test/chi square** |
| --- | --- | --- | --- | --- |
| **Cohort characteristics**  Years of follow-up – mean (SD)  Deaths: n (%) | 4.5 (1.6)  58 (31.7%) | 5.2 (1.1)  707 (13.5%) | 4.9 (1.1)  765(14.1%) | <0.001  <0.001 |
| **Sociodemographic data**  Age at baseline – mean (SD)  Female gender: n (%)  Married/cohabiting: n (%)  Years of education – mean (SD)  Skin color (non white) n (%)  Urban residence | 77.1 (9.6)  116 (63.4)  64 (35.0)  1.7 (2.4)  114 (62.3)  137 (74.9) | 70.1 (7.8)  3,144 (59.9)  2,723 (51.9)  4.5 (4.1)  2,931 (55.8)  4,416 (84.1) | 70.3 (8.0)  3,260 (60.0)  2,787 (51.3)  4.4 (4.1)  3,045 (55.8)  4,553 (83.8) | <0.001  0.343  <0.001  <0.001  0.08  0.001 |
